# The role of microglia in neuropsychiatric disorders and suicide

**DOI:** 10.1007/s00406-021-01334-z

**Published:** 2021-09-30

**Authors:** Ralf Brisch, Szymon Wojtylak, Arthur Saniotis, Johann Steiner, Tomasz Gos, Jaliya Kumaratilake, Maciej Henneberg, Rainer Wolf

**Affiliations:** 1 Department of Forensic Medicine, Medical University of Gdańsk, Gdańsk, Poland; 2 Department of Pathomorphology, Medical University of Gdańsk, Gdańsk, Poland; 3 Department of Anthropology, Ludwik Hirszfeld Institute of Immunology and Experimental Therapy, Polish Academy of Sciences, Wroclaw, Poland; 4 Department of Pharmacy, Knowledge University, Erbil, Kurdistan Region Iraq; 5 Department of Psychiatry and Psychotherapy, Otto-von-Guericke-University, Magdeburg, Germany; 6 Biological Anthropology and Comparative Anatomy Research Unit, Medical School, The University of Adelaide, Adelaide, Australia; 7 Institute of Evolutionary Medicine, University of Zurich, Zurich, Switzerland; 8 Department of Nursing and Health, Hochschule Fulda, University of Applied Sciences, Fulda, Germany

**Keywords:** Microglia, Schizophrenia, Major depressive disorder, Bipolar disorder, Affective disorders, Suicide, Dorsal raphe nucleus, Serotonin, Evolution of human lineage

## Abstract

This narrative review examines the possible role of microglial cells, first, in neuroinflammation and, second, in schizophrenia, depression, and suicide. Recent research on the interactions between microglia, astrocytes and neurons and their involvement in pathophysiological processes of neuropsychiatric disorders is presented. This review focuses on results from postmortem, positron emission tomography (PET) imaging studies, and animal models of schizophrenia and depression. Third, the effects of antipsychotic and antidepressant drug therapy, and of electroconvulsive therapy on microglial cells are explored and the upcoming development of therapeutic drugs targeting microglia is described. Finally, there is a discussion on the role of microglia in the evolutionary progression of human lineage. This view may contribute to a new understanding of neuropsychiatric disorders.

## Introduction

Microglial cells originate from myeloid precursors in the yolk sac and are regarded as resident macrophages of the central nervous system (CNS) [1]. During the development of the CNS, microglial cells have various functions: they phagocytose apoptotic neurons, and induce neuronal apoptosis, prune weak synapses, form new synapses, promote the survival of pyramidal neurons in the white matter, direct the expression of (S)-2-amino-3-(3-hydroxy-5-methyl-4-isoxazole)-propionic acid—(AMPA) and N-methyl-D-aspartate—(NMDA) receptors at thalamocortical synapses and regulate the production and migration of cortical inhibitory neurons [1]. Activated microglia produce cytokines and nitric oxide (NO). Activated microglial cells are polarized towards the M1 or M2 phenotype. While polarization towards the M1 phenotype, also called the proinflammatory phenotype, is caused by cytokines such as interferon-alpha (IFN-α) and tumor necrosis factor-alpha (TNF-α), polarization towards the M2 phenotype, also termed the anti-inflammatory phenotype, is triggered by various cytokines such as interleukin IL-4, IL-13 and IL-25 [2–11]. The differences between the M1 and M2 phenotypes have been critically discussed and might be revaluated in the future [10, 12].

## Microglial cells and neuroinflammation

Neuroinflammation is inflammation of nervous tissue [13]. The hallmark of neuroinflammation in schizophrenia (SZ) is overactivation of microglial cells [14]. Acute neuroinflammation during fetal development causes neuropathological abnormalities in the cerebellum, insular cortex, fusiform gyrus and deficits in neuronal activation in the right amygdala and fusiform gyrus, and ventrolateral prefrontal cortex (PFC) [15]. Neuroinflammation is mainly facilitated by microglial cells and to same extent by astrocytes and mast cells [16]. Microglial cells are divided into amoeboid and ramified cells. The so-called dark microglial cells, which are highly phagocytic cells under oxidative stress around the vasculature showing an electron-dense, condensed cytoplasm, are either overactivated microglial cells or a novel type of myeloid cell infiltrating the brain [16]. Neuroinflammation is linked to white matter pathology in patients with schizophrenia and is caused by microglial cells [17, 18]. Furthermore, focal neuroinflammation occurs in the hippocampi of patients with schizophrenia with an acute psychosis [19]. Disruption of the interaction between mast cells and microglial cells increases neuroinflammation [20]. However, the term neuroinflammation is not interchangeable with the term inflammation: microarray analyses of postmortem cerebral cortices of patients with Alzheimer’s disease (DAT), Parkinson’s disease (PD), patients with schizophrenia and patients with inflammatory diseases have demonstrated no relationship between these conditions [21].

When microglial cells become activated during neuroinflammation, they become enlarged and phagocytic. Microglial cells contribute to synaptic plasticity by releasing neuroactive molecules such as adenosine triphosphate (ATP), glutamate, D-serine, nitric oxide (NO), brain-derived neurotrophic factor (BDNF), TNF-α, free radicals, prostaglandin E2 (PGE2), and ILs; and microglial cells communicate with astrocytes through glutamatergic neurotransmission [2]. Furthermore, glutamate controls the function of microglial cells via ionotropic and metabotropic receptors located in these cells [22]. Microglial cells also communicate with neurons, promoting neuronal cell death, neurogenesis, and synaptic interactions [7, 23, 24]. Activated microglia control inhibitory inputs from parvalbumin containing interneurons onto the basilar dendritic spines of deep layer 3 pyramidal neurons in the PFC in patients with schizophrenia [25]. Microglia–neuronal interactions involve various signals such as cytokines, neurotransmitters, and neuron-microglia inhibitory factors, such as fractalkine (CXCL1) and cluster of differentiation (CD200) [26, 27]. Microglial cells are also connected with astrocytes and oligodendrocytes, and these interactions might also cause neuropathic pain [28]. Dystrophic microglial cells contribute to substantial dystrophies in adjacent oligodendrocytes in the PFC (layer 5) in patients with schizophrenia with predominately negative symptoms and to a lesser extent in patients with schizophrenia with mainly positive symptoms but not in healthy control subjects [29]. Disruption of the balance between microglial cells and astrocytes (e.g., type-1/type-2 imbalance) causes a dysregulation of the immune response in schizophrenia [30, 31]. T-helper 1 cells (TH-1) and certain macrophages/monocytes (M1) produce cytokines (IL-2, IL-12, IL-18, IFN-α, TNF- α) and this immune response is called type-1 immune response. T-helper 2 cells (TH-2) and certain macrophages/monocytes (M2) create cytokines (IL-4, IL-10, IL-13) and this immune response is termed type-2 immune response. Type-1 and type-2 cytokines irritate each other with a down-regulation in schizophrenia [30].

## The various roles of microglial cells in neuropsychiatric disorders

Microglial cells play an important role in the pathology of neuropsychiatric disorders such as schizophrenia and major depressive disorder (MDD) [31–35]. Microglial cells are also involved in the cerebral cortex with autism spectrum disorders (ASDs), which are also regarded as neurodevelopmental disorders [36, 37]. Activated microglia and neuroinflammation are also pathological hallmarks of neurodegenerative diseases such as DAT and PD [38]. Original research on postmortem and PET studies demonstrating the presence or absence of microglial activation using different microglial markers in brain regions of interest, predominantly, in patients with schizophrenia, bipolar disorder, and affective disorders, respectively, is summarized in Table 1.

**Table 1:** Postmortem and PET studies demonstrating the presence or absence of microglial activation using different microglial markers in brain regions of interest in patients with schizophrenia, bipolar disorder and affective disorders

Study	Subjects	Methods	Brain regions	Results
Bayer et al. [53]	14 SZ,	Histology, HLA-DR	HPC, PFC	Activation of HLA-DR ↑
	8 AD			
	13 CTR,			
Radewicz et al. [54]	8 chronic SZ, 10 CTR (area 9), 7 chronic SZ, 6 CTR (area 22)	Histology, HLA-DR	DLPFC (Brodmann`s area 9), STG (area 22), ACC (area 24)	Area 9 ↑,
				Area 22 ↑
Wierzba-Bobrowicz et al. [55]	9 SZ, 6 CTR	Histology, HLA-DR	Frontal and temporal cortices	HLA-DR ↑
Steiner et al. [57]	16 SZ	Histology, HLA-DR	DLPFC, ACC, MD	DLPFC ↔
	16 CTR			ACC ↔ , MD ↔ , cerebral lateralization of amoeboid microglia in SZ ↓, ↑ ACC, ↑ MD in SZ, who committed suicide
Steiner et al. [52]	16 SZ	Histology, HLA-DR	DLPFC, ACC, MD, HPC	Significant differences in microglial cell densities ↔ among the groups; significant increases ↑ in DLPFC, ACC and MD in patients, who committed suicide
	14 AD			
	10 CTR			
Doorduin et al. [19]	7 SZ	Pet with ^11^C-(R)-PK1195	Frontal, occipital, temporal, parietal lobes, basal ganglia, thalamus, HPC, midbrain, cerebellum, pons	Binding of ^11^C-(R)-PK1195 ↑ in the HPC in SZ
	8 CTR			
Busse et al. [59]	17 SZ	Histology, CD3 + T-lymphocytes, CD20 + B-lymphocytes, HLA-DR + microglia	Posterior HPC	CD3 + , CD20 + ↑ in residual SZ versus paranoid SZ, HLA-DR ↑ in paranoid SZ versus residual SZ
	11 CTR			
Fillman et al. [58]	37 SZ	Histology, HLA-DR	DLPFC	Density in the white matter ↑ in SZ, density in gray matter ↔ in SZ
	37 CTR			
Kenk et al. [188]	16 SZ	PET with TSPO	Gray matter: striatum, HPC, PFC, DLPFC, TC, white matter: CC, CING, SLF, PLIC	Significant difference in neuroinflammation ↔
	27 CTR			
Najjar and Pearlman [17]	15 studies: 350 SZ, 49 BD, 37 MDD, 10 AD, 346 CTR	Systematic review, neuropathological and neuroimaging studies	Gray and white matter	Microglial activation in the white matter in SZ,
Busse et al. [175]	12 AD (6 MDD, 6 BD)	Histology, microglial QUIN	HPC (CA1, CA2/3 areas)	QUIN ↓ in the right CA1 area in MDD and BD
Bloomfield et al. [45]	14 ultra-high risk SZ	PET with TSPO	Total gray matter, volume, frontal and temporal lobe gray matter	Microglial activity ↑ in ultra-high risk SZ
	14 CTR			
Di Biase et al. [47]	10 ultra-high risk SZ	PET with TSPO	Dorsal frontal, orbital frontal, medial temporal cortex, medial temporal cortex, ACC	Significant difference in TSPO expression ↔ , microglial activation ↔
	18 patients recently diagnosed with SZ			
	15 chronic SZ			
	27 CTR			
Brisch et al. [104]	18 SZ	Histology, HLA-DR	DR	Microglial reaction ↓ in nonsuicidal patients compared with suicidal patients and CTR
	15 MDD			
	12 BD			
	22 CTR			
Hafizi et al. [46]	14 first-episode SZ	PET with TSPO	DLPFC, HPC, medial prefrontal and temporal cortices, total gray matter	Significant difference in TSPO expression ↔ , microglial activation ↔
	20 CTR			
van Kesteren et al. [41]	783SZ	Meta-analysis, histology, various microglial markers	TC, PFC, OC, diencephalon, basal ganglia, ACC	Significant increase in microglial cell density ↑
	762 CTR			
Conen et al. [49]	20 ROS	PET with TSPO	ACC, PFC, PC, putamen, thalamus, and brainstem	Microglial activation ↔ in ACC, PFC, PC, and brainstem; microglial activation ↓ in thalamus and brainstem in ROS and ES
	21 ES			
	21CTR			
Petrasch-Parwez et al. [44]	17 SZ	Histology, Iba-1	aMC	No significant differences in IBA1 microglial densities between SZ, BD, and controls. A lateralization towards the right anterior midcingulate cortex was obsevered in SZ, BD, not in controls
	13 BD			
	17 CTR			

Abbreviations: *ACC* anterior cingulate cortex, *AD* patients with affective disorders, *aMC* anterior midcingulate cortex, *BD* patients with bipolar disorder, *CC* corpus callosum, *CING* cingulum, *CTR* healthy control subjects, *DAT* patients with dementia of Alzheimer’s type, *DLPFC* dorsolateral prefrontal cortex, *DR* dorsal raphe nucleus, *ES* patients with established schizophrenia, *HPC* hippocampus, *HLA-DR* human leukocyte antigen, *MD* mediodorsal thalamus, *MDD* patients with major depressive disorder, *OC* occipital cortex, *PC* parietal cortex, *PET* positron emission tomography, *PFC* prefrontal cortex, *PLIC* posterior limb of the internal capsule, *QUIN* quinolinic acid, *ROS* patients with recent onset schizophrenia, *SLF* superior longitudinal fasciculus, *TSPO* translocator protein, *TC* temporal cortex

Arrows: ↑ increase, ↔ no alteration ↓ decrease

### The role of microglial cells in schizophrenia

Glucocorticoid levels are increased during stress, and influence microglial response with pro- and anti-inflammatory activity [9]. Stress-induced release of glutamate from neurons results in the stimulation of microglial cells through the activation of N-methyl-D-aspartate receptors (NMDARs) by glucocorticoids [8, 9].

Microglial cells induce the loss of cortical gray matter in patients with schizophrenia by pruning synapses, phagocytosing stressed neurons, and preventing the release of neurotrophic factors such as BDNF [39].

In a systematic review by Trépanier et al. [40], many of the studies (11 of 22) reported an increase in the expression of microglial markers in the postmortem brains from patients with schizophrenia compared with those of control subjects. Moreover, a meta-analysis by van Kesteren et al. [41] revealed an increase in the number of microglia in specific brain areas, such as the temporal cortex, in postmortem brains of patients with schizophrenia compared with those of control subjects. Calprotectin is expressed in microglial cells and is considered a nonspecific marker of inflammation. Calprotectin expression is significantly increased in patients with schizophrenia compared with healthy control subjects [42]. The level of ionized calcium-binding adaptor molecule 1 (IBA1), a marker showing specific immunostaining of resting and activated microglia, is increased in specific brain areas such as the amygdala, hippocampus, nucleus accumbens, and PFC in patients with schizophrenia [9, 43]. A lateralization of IBA1 immunopositive microglial cells was observed towards the right anterior midcingulate cortex in patients with schizophrenia and bipolar disorder compared with healthy control subjects [44]. A study by Bloomfield et al. [45] using translocator-protein positron emission tomography (TSPO PET) demonstrated that microglial activity is increased in total and frontal and temporal lobe gray matter in patients with schizophrenia and ultrahigh risk individuals compared with healthy control subjects. Nevertheless, an 18 kDa TSPO PET study revealed no increase in microglial activity in the dorsolateral PFC and hippocampus in first-episode psychosis patients compared with healthy control subjects [46]. Moreover, another TSPO PET study by Di Biase et al. [47] did not show any differences among individuals at risk for schizophrenia, patients with schizophrenia and healthy control subjects. A meta-analysis by Marques et al. [48] of TSPO PET studies and microglial activation exposed a moderate effect on gray matter relative to other brain tissue in schizophrenia when using binding potential as an outcome measure but no difference when using volume of distribution as an outcome measure. The TSPO PET study by Conen et al. [49] found no microglial activation in patients with recent onset and established schizophrenia compared with healthy control subjects in the ACC, PFC, parietal cortex, and brainstem, but significant changes in the thalamus and putamen.

Activated microglial cells are linked to the increased expression of peripheral benzodiazepine binding sites (PBBS). (R)-PK1195 is a specific ligand for the PBBS and combines with carbon-11 in PET studies in schizophrenia, which can be used as a novel PET biomarker of activated microglial cells in schizophrenia [50].

The discrepancies in findings related to microglia activation in schizophrenia obtained by PET- and postmortem studies may have been caused by effects of confounding factors (e.g., tissue quality and aging) and comorbid factors (e.g., use of antipsychotic medication, suicidal tendencies, smoking, or drug abuse) [24].

Human leukocyte antigen (HLA-DR) is a class II antigen, and different gene loci code for the alpha (α)- and beta (ß)-chains. Other important genes, namely, TNF-α and tumor necrosis factor beta (TNF-ß) are distributed throughout the HLA-complex [51]. Steiner et al. [52] (2008) found that the density of HLA-DR, which reacts with activated microglia, was increased in the PFCs of suicidal patients. Bayer et al. [53] reported that three late-onset out of 14 patients with schizophrenia, 1 out of 6 patients with affective disorders (AD), and four out of 8 patients with dementia of Alzheimer’s type (DAT) exhibited increased HLA-DR immunostaining in the frontal cortex and hippocampus. Radewicz et al. [54] observed an increase in HLA-DR density in the frontal and temporal cortices in patients with schizophrenia compared with control subjects, and Wierzba-Bobrowicz et al. [55] detected a greater expression of HLA-DR in the anterior cingulate and temporal cortices in patients with schizophrenia compared with control subjects. Additionally, Krause et al. [56] found that the number of HLA-DR immunopositive cells was increased in patients with schizophrenia compared with healthy control subjects. Steiner et al. [57] reported decreased cerebral lateralization of HLA-DR in patients with schizophrenia compared with healthy control subjects. Fillman et al. [58] found an increased HLA-DR in the dorsolateral prefrontal cortex in patients with schizophrenia compared with control subjects. A study by Busse et al. [59] also demonstrated that HLA-DR immunostaining was increased in the posterior hippocampus in patients with paranoid schizophrenia compared with patients with residual schizophrenia and healthy control subjects. Moreover, patients with residual schizophrenia showed a greater concentration of CD3^+^ and CD20^+^ lymphocytes in the posterior hippocampus [59]. No significant differences in microglial density using the microglial marker IBA1 in the medial frontal gyrus in patients with bipolar disorder compared with healthy control subjects have been demonstrated [60]. A correlation has also been found between HLA-DR and alcoholism or even alcoholism withdrawal [61–64].

These discrepancies in the results of postmortem studies could be explained by methodological differences such as the immunohistochemical markers used, the methods used to count microglial cells, the brain regions studied, the cortical layers in which the expression of the markers was measured, and technical issues such as the postmortem interval and brain pH. Other confounding variables including age differences, sex, age at death, cause of death, clinical variables, illness history and duration, medication history, diagnosis method and inclusion and exclusion criteria also exist [14, 65]. Recently, microglial activation rather than microglial density has been investigated [65]. Microglial activation reflects innate immune memory (history of life events) including prenatal and perinatal influences and genetic background [14, 66]. Furthermore, the use of a single microglial marker (such as HLA-DR) for phenotypic identification is problematic, since detailed knowledge of the time course of death is crucial [14]. Multiple markers must be used to identify the functional state [14]. The association between microglial activation and reduced synaptic plasticity, and neuropil alterations and reduced neuroplasticity needs to be studied in a multimodal manner combining imaging studies and postmortem brain analyses of patients with schizophrenia with various immunological markers [14].

Research conducted by Goudriaan et al. [67] has demonstrated that genes that are involved in microglial activation do not contribute genetically to schizophrenia susceptibility. However, there is an association between the HLA-DRB1 gene and schizophrenia in the human population [68–70].

Maternal infection during pregnancy is known to be an environmental risk factor for the development of neuropsychiatric disorders such as schizophrenia and ASDs in offspring. The causative role of immunological processes that interfere with brain development in schizophrenia have been discussed [71–73]. Sex differences in processes related to microglial cells exist and might contribute to the sex differences in neuropsychiatric disorders such as schizophrenia [74, 75] and in a neonatal rat animal model of early-life infection [76].

Although Smolders et al. [77] demonstrated that a single or double injection of poly(I:C) in pregnant mice does not result in a fetal microglial activation during mid- or late embryonic development, other research groups have found an involvement of microglial cells in maternal immune activation. In an animal study using pregnant mice injected with poly(I:C) on gestational day 9 as an animal model of schizophrenia, the number and shape of microglia in several brain regions were assessed in the offspring on postnatal day (PND) 30 using immunofluorescence with an anti-IBA1 antibody [78]. There were more microglial cells in the hippocampus and striatum in the offspring of poly(I:C)-injected mice than in the offspring of vehicle-treated mice. Additionally, these microglial cells were morphologically characterized by reduced arborization, which is indicative of a greater activation. This is supported by studies showing that microglial cells from control animals show greater microglial arborization, which is indicative for a noninflammatory state of microglia than those from offspring from poly(I:C) treated mice, which were characterized by no or few branched cells, indicating an activated and inflammatory or phagocytic microglial state [79, 80]. Furthermore, in a pilot study comparing the offspring of poly(I:C) treated mice on PND10 and PND100, the mouse pups displayed no microglial alterations in response to prenatal exposure to poly(I:C), whereas adolescent mice showed a significant increase in the number of microglial cells in the frontal association cortex, ventral striatum, dentate gyrus of the hippocampus and secondary visual cortex. In contrast, in adult mice (PND100), immunological activation was only observed in the frontal association cortex [81]. Prenatal poly(I:C) treatment in mice prevents an increase in the number of microglial cells in the cortex [82]. These studies support the hypothesis that maternal infection during embryogenesis contributes to an increase in the number of microglial cells in offspring, which in subsequent periods of life results in decreases in dopamine and the stimulation of high-affinity dopamine receptors (involving DRD3 and DRD5). These alterations in dopamine levels may, therefore, cause a neuroinflammatory response, which contributes to the pathogenesis of schizophrenia [83].

### The involvement of microglial cells in depression and suicide

Suicide is a major public health problem [84] with a prevalence of 5.92% and is supported by ongoing neurobiological research [85], suicide behavior is listed as an independent mental disorder in the fifth edition of the Diagnostic Statistical Manual of Mental Disorders-DSM V [86]. The dorsal raphe nucleus (DRN) comprises the caudal and rostral subregions, which are further divided into the dorsal, ventral, interfascicular, ventrolateral, and caudal subregions [87]. The DRN sends serotonergic projections to the striatum, frontal cortex, thalamus, lateral septal nuclei, nucleus accumbens, habenular complex, and hippocampus [88–93]. The dorsal raphe nucleus is implicated in the pathology of schizophrenia, major depression, suicidal behavior [94–104] and even alcoholism [105]. Disruption of serotonergic functions is implicated in the pathophysiology of stress, MDD, and suicide [106–110], while serotonin deficiency contributes to suicidal behavior and MDD [111–126]. Additionally, a link between smoking, suicide, and low serotonin levels has been demonstrated [127]. Nicotine-induced microglial activation in reward seeking brain regions such as nucleus accumbens and basolateral amygdala increases cocaine reinforcement in adolescent rats but not adult rats [128].

Microglial cell activation correlates with transcriptional activity in the DRN neurons in the non suicidal depressed subgroup [104]. In addition, microglial activation induced by interferon-alpha (IFN-α) is associated with depressive-like behavior and the expression of proinflammatory surface markers such as major histocompatibility complex-II (MHC-II) and CD86 in a mouse model of immune-mediated depression, demonstrating that microglia are polarized towards the M1-phenotype [129]. A study by Steiner et al. [52] demonstrated that microglial density is increased in the dorsolateral prefrontal cortex, anterior cingulate cortex, and mediodorsal thalamus in suicidal patients with schizophrenia and suicidal patients with depression compared with healthy control subjects. Schnieder et al. [130] also observed that microglial density is augmented in the white matter of the PFC in suicidal patients with schizophrenia and depression compared with nonsuicidal subjects. Various studies (e.g., postmortem analysis, PET imaging studies, and analysis of the cerebrospinal fluid and serum/plasma of patients) have provided evidence for a role of microglial cells in suicide, which can be used for a new therapeutic target for suicide prevention [131]. Adolescence is a significant period for the development of neuropsychiatric diseases. During this period microglial synaptic pruning occurs in the PFC and contributes to pathological alterations that are evident in schizophrenia [132]. An increase in the number of microglial cells leads to depression through the neuroinflammatory pathway. Activated microglia are also involved in post-stroke depression [133]. Additionally, a decrease in the number of microglial cells causes depression by inducing neuronal degeneration, leading to inhibition of neurogenesis in the hippocampus [134].

Most studies have demonstrated an association between suicidal behavior and alterations in IL-2, IL-6, IL-8, TNF-α and VEGF levels [135]. Elevation of IL-6 levels is one of the most prominent findings in patients exhibiting suicidal behavior. However, future research should focus on the association between cytokines, suicidal behavior, and depression [136, 137]. Moreover, the number of primed microglial cells is increased in the white matter of the dorsal anterior cingulate cortex in depressed suicidal patients compared with healthy control subjects [138].

In an animal model of depression, prenatal stress may be linked to increased activity of microglial cells in the hippocampus and frontal cortex and depression-like disturbances [139, 140]. The hippocampus, especially the CA1 region, is characterized by an elevated number of microglial cells, which are activated by stress [7, 141]. The adult microglial transcription factor MafB is involved in the expression of the adult gene program during inflammatory responses under stress as demonstrated by experiments with knockout mice [142]. Another brain region that is affected by microglial cell activation is the postnatal amygdala, which is involved in emotion, as maternal immune activation causes the activation of microglial cells in the amygdala [143].

The neuroprotective role of microglial cells has been extensively discussed [144–147]. For example, microglial cells exert neuroprotective effects through anti-inflammatory responses in depression [148, 149] and schizophrenia [150]. Microglia also have neuroprotective effects after ischemia [151]. On one hand, microglial cells are responsible for maintaining neuronal structure and plasticity via clearance of cellular debris, neurogenesis, anti-inflammatory responses, and synaptic pruning [149]. On the other hand, neurons play a vital role in the functions of microglial cells by maintaining inflammatory gene production, the oxidative stress response, and synaptic pruning [149, 152]. Therefore, if the balance between microglial cells and neurons is disturbed, neuropsychiatric disorders may result [149, 153–155]. Microglial cells have also been demonstrated to play a neurodegenerative role [156]. For example, a disturbance of the interplay between microglial cells and cytokines may cause neurodegenerative processes in neuropsychiatric diseases such as schizophrenia [34]. Peripheral blood mononuclear cells were combined with neural progenitor cells and pluripotent stem cells to produce microglia-like cells, which express specific markers and function as microglial cells [157]. Microglial cells and monocytes interact with each other to regulate the expression of genes, cytokines, and surface markers through the blood–brain barrier and neuronal networks, and these interactions might be useful for the developing peripheral biomarkers for psychiatric disorders [157].

Prenatal immune challenge by poly(I:C) causes microglial cell alterations in different structures such as microglial clusters, including changes in morphology, the arborization index and the number of dark microglia in the dentate gyrus of the hippocampus, mainly in male mice. These changes are features of cellular stress [158]. Hinwood et al. [159] observed that microglia facilitate the effects of stress on neurons in the medial PFC. Stress exposure correlates with increased microglial activation in the PFC but not antigen presentation [159]. Similarly, microglia are responsible for the impact of stress on neuronal branching in the PFC. Treatment with minocycline eliminates these effects [160]. In patients with depression, repeated stress exposure can lead to microglial inflammation or even suicidal behavior [161]. It has been shown that increased apoptosis causes a reduction in microglial cell number [26].

In the CNS, the kynurenine (KYN) pathway is affected by astrocytes, microglial cells, and macrophages. KYN can be converted to kynurenic acid or quinolinic acid [162–164]. Abnormal KYN levels are implicated in neurodevelopmental disorders through the proliferation, specification, and differentiation of radial glial cells [165] and the pathophysiology of schizophrenia and affective disorders [166–171]. By exploring the inflammatory process and hence the kynurenine pathway, novel therapeutic targets and biomarkers for suicidal depressed patients [172] and patients with schizophrenia [173] might be developed. For example, Steiner et al. [174] reported an increase in the level of quinolinic acid, which is produced by microglial cells, in subregions of the anterior cingulate gyrus in depressed patients. Furthermore, investigations by Busse et al. [59, 175] revealed that quinolinic acid levels are decreased in the hippocampus of suicidal depressed patients compared with healthy control subjects and in patients with paranoid schizophrenia compared with patients with residual schizophrenia [176].

Early postnatal lipopolysaccharide (LPS) administration in a rat animal model of early immune stimulation causes a reduction in hippocampal volume, activation of the KYN pathway of tryptophan metabolism, astrogliosis, and a decrease in tyrosine hydroxylase levels in the substantia nigra demonstrating a link between early immune stimulation and neuropsychiatric disorders [177]. LPS-induced maternal immune activation causes an increase in cytokine levels in the fetal brain, which results in elevation of microglial activation, astrogliosis and cytoarchitectural changes in the postnatal amygdala [143].

## The effects of antidepressants, antipsychotics, and electroconvulsive therapy (ECT) on microglial cells

The long-term influences of antidepressants and antipsychotics on microglial cells in various brain regions should not be ignored, as they might offer new options for drug treatment. Therefore, we will discuss the effects of antidepressants, antipsychotics, and electroconvulsive therapy on microglial cells. Typical and atypical antipsychotics suppress microglial activity by inhibiting the secretion of cytokines [178]. Specific interleukins (Il-10, IL-10, and TGF-α) are state markers and increase during acute phases of schizophrenia, but are normalized with antipsychotic medication. Both IL-12, IL-2, interferon- α, and TNF-α are characterized as trait markers and continue to be high during acute phases independent of antipsychotic medication [179]. Typical and atypical antipsychotics influence the intracellular Ca^2+^ mobilization and signaling in the endoplasmic reticulum (ER) of microglial cells in different ways. Thus, these processes might be targets for antipsychotic therapy [180].

Specifically, inhibitors of microglial activity such as minocycline are regarded as potential antipsychotic drugs [181–183]. Riazi et al. [184] showed that minocycline decreased the effects of inflammation and abolished the effects of peripheral inflammation in the hippocampi of rats. For example, minocycline selectively inhibits the expression of M1-polarized microglia in vivo and in vitro in amyotrophic lateral sclerosis (ALS) [185].

Cotel et al. [186] reported that chronic antipsychotic use increases microglial activation and proliferation in the rat brain. Cotel et al. [186] revealed that antipsychotic treatment not only reduced the gray matter volume in the hippocampus, anterior cingulate cortex, corpus striatum, and secondary somatosensory cortex but also increased the density of microglial cells in the hippocampus and somatosensory cortex but surprisingly not in the anterior cingulate cortex. The reason why the microglial density was not increased in the anterior cingulate cortex by chronic antipsychotic use requires further research. In rat experiments, clozapine was shown to increase microglial activation in the striatum and hippocampus, mainly the CA2 and CA3 regions [187]. In contrast, antipsychotics inhibit the release of proinflammatory cytokines and NO by microglial cells [188, 189]. In summary, microglial activity plays a role in the pathophysiology of schizophrenia and anti-inflammatory drugs such as minocycline might be useful for targeting the increased microglial activation in schizophrenia and thus be new treatment options [190, 191]. Regulation of microglial activation site-specifically and function-specifically might be a novel target for the development of new drugs for patients with schizophrenia [192, 193].

**Fig. 1:**
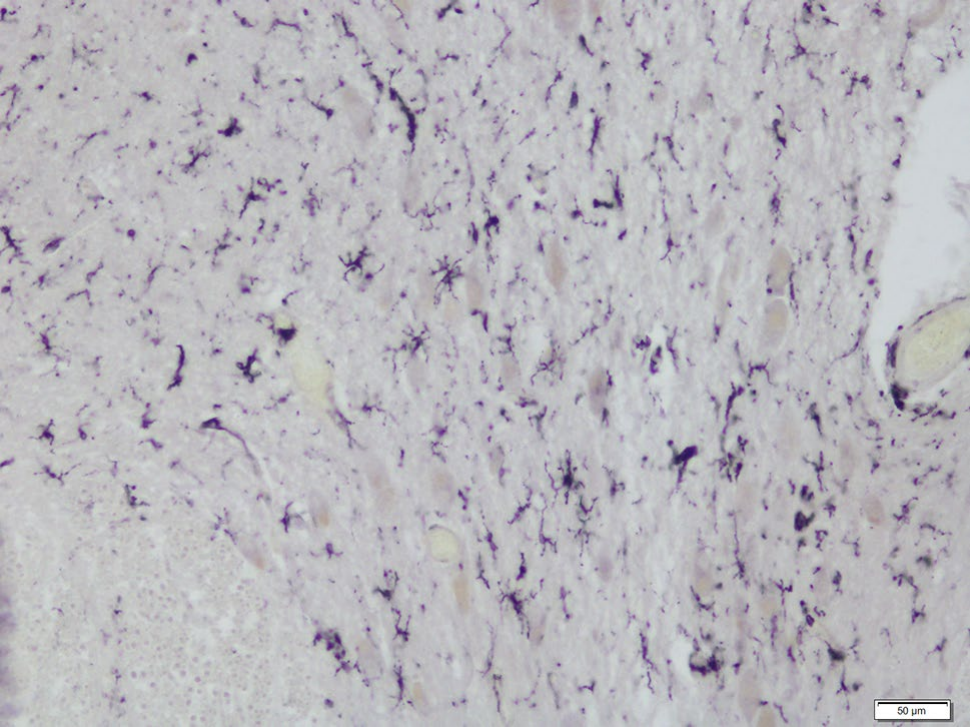
Microglial reaction in the DRN (caudal subdivision) in suicidal depressed patient as shown by HLA-DR antigen, scale bar 50 µm

**Fig. 2:**
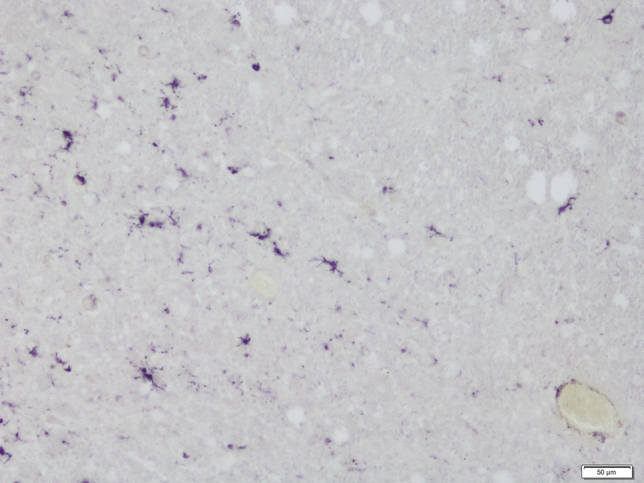
Microglial reaction in the DRN (caudal subdivision) in non-suicidal depressed patient as shown by HLA-DR antigen, scale bar 50 µm

For example, studying single-cell screening from blood serum taken from drug-naïve patients with schizophrenia the phenotypic modification of microglial cell signaling in vitro was demonstrated [193]. Overactivated epitopes in blood sera of patients with schizophrenia can be ameliorated by microglial proinflammatory activation inhibitors such as minocycline to improve negative symptoms in schizophrenia [194]. Antagonists of the ATP-gated P2X7 receptor, which is a microglial receptor found in the CNS, might be used as monotherapies or adjunct therapies for the treatment of schizophrenia, bipolar disorder or depression [194]. Activated microglia can be used as biomarkers for predicting the course in bipolar disorder and pharmacological responses [195]. Stertz et al. [196] argued that the inflammatory changes that occur in bipolar disorder patients are associated with disease progression rather than causative. Additionally, the selective serotonin reuptake inhibitor (SSRI) fluoxetine protects neurons against the neurotoxic effects of microglial cells [197]. SSRIs increase TNF-α levels, and when used at low concentrations for a long period of time, SSRIs have slight proinflammatory effect [198]. In contrast, Horikawa et al. [199] reported that SSRIs inhibit the production of NO and TNF-α in microglial cells. Additionally, the SSRI fluoxetine and its metabolite norfluoxetine reduce the viability of microglia and increase the expression of the apoptotic marker cleaved-caspase 3 in microglial cells [200]. Depression is also considered a microglial disease and drugs that either suppress or stimulate microglial cells are regarded as novel drugs in the treatment of depression [147]. Various antidepressants and antipsychotics overturn microglial activation. Antidepressants inhibit the release of proinflammatory cytokines from activated microglial cells [201]. The antidepressant drug venlafaxine can partially protect the viability of microglial cells [202]. Antidepressants cause microglial cell disturbances in the dorsal raphe nucleus [104]. A positive correlation between antidepressants and microglial density in non-suicidal depressed patients in the dorsal raphe nucleus has been reported [104] (Figs. 1 and 2).

Glutamate has neurotoxic effects on activated microglial cells [203]. For example, an inhibition of glutamine synthetase increases the activity of activated microglial cells [204]. However, a general blockade of microglial cells as a therapeutic intervention might have risky effects, since glutamate and activated microglial cells are involved in dendritic apoptosis. An excessive synaptic pruning during late adolescence and early adulthood might cause severe negative symptoms and a cognitive decline in patients with schizophrenia [205, 206].

Additionally, the use of induced microglial cells (iMG) from the peripheral blood [207] and the use of human-induced pluripotent stem cells for microglial cell culture methods [208] might represent novel directions for psychopharmacological screening or schizophrenia modeling. Cannabis and cannabidiol reduce the oligodendrocyte, astrocyte, and microglial activity in schizophrenia [209, 210]. For example, activated cannabinoid type -2 (CB2) receptors located on microglial cells decrease the effects of activated microglial cells such as neuroinflammation and synaptic pruning [211]. ECT either reduces microglial cell density in the hippocampus [212] or activates microglial cells [213]. It has been demonstrated that microglial cell activation in the hippocampus is inhibited by ECT in a rat model [214].

## Evolutionary role of microglial cells in neuropsychiatric disorders

Microglia probably arose during the Cambrian period (500–540 Ma ago) with the propagation of multicellular animals. Microglia have been evolutionarily conserved in both invertebrates and vertebrates [215–217]. It has been argued that early microglia contributed to the protection of the CNS in emerging vertebrates, and informed axonal coverage, thus increasing action potential transmission and speed and thereby improving CNS efficiency [218].

It is worth noting that human neuronal density is much lower than that of chimpanzees and other apes and primates [219]. This means a greater volume of glial cells, likely also microglia in proportion to neurons. Such an arrangement allows for greater role of glial cells in processing of information in the human brain. It has been shown that working memory is different in humans than in chimpanzees. In humans, there is evidence that genes of abnormal spindle-like microcephaly-associated protein (APSM), neuronal cell adhesion molecule (NRCAM) and sialic acid-binding Ig like lectin 11 (SIGLEC11) are involved in neural proliferation, promotion of neural connections, and glial expression, respectively [220, 221]. There is a difference in ASPM and SIGLEC-11 in the human lineage expression compared to chimpanzee lineage. SIGLEC-11 is a human-specific microglial gene that is not found in other primates [222]. A study by Wang et al. [223] using human brain tissue demonstrated that human SIGLEC-11 ectopically expressed by a lentiviral vector system in cultured murine microglial cells interacts with polysialic acid (PSA) residues on neurons, reduces LPS-induced gene transcription of proinflammatory mediators, impairs phagocytosis and alleviates microglial neurotoxicity.

The pathogen host defence (PATHOS-D) theory suggests that the immune responses that occur in MDD are proinflammatory and that specific risk alleles for MDD generate a proinflammatory response, which is a driving force in human evolution [224]. Divergence from microglial stability due to inflammatory conditions that trigger microglial activity (e.g. infections, neurodegenerative diseases, stress, brain trauma) are risk factors for depression [147].

It should be noted that during the last 2 million years there have been notable changes in the neurohormonal regulation of the human brain due to self-amplifying feedback processes that contributed not only to the size and capacity of the modern human brain but also to its susceptibility to psychopathologies. *H. erectus* (1–8 Ma) exhibited a marked increase in physical activity levels (PALs) and total energy expenditure (TEE) compared to australopithecine and the great ape lineages [225, 226]. Selective processes enhanced aerobic mechanisms and modified the thermoregulatory response (i.e., hairlessness in humans facilitated evaporative sweating and increased UV exposure) [227, 228] to optimize the bodies of archaic hominins for persistent hunting in hot environments, thereby increasing the production of growth factors, such as BDNF [229, 230]. Selection for characteristics important for persistent hunting and endurance is biased towards muscle composition changes and the development of more efficient energy systems involving peroxisome proliferator-activated receptor γ coactivator (PGC-1α) and myocyte enhancer factor-2 (MEF2) gene regulation, which stimulate BDNF factor activity [227]. In archaic hominins, BDNF informed brain evolution in cortical and subcortical areas [231, 232]. BDNF also contributes to neurogenesis in the hippocampus, memory and learning. Synaptic plasticity is associated with prenatal brain development [233, 234] and neuroprotection, and ~ 80% of synaptic plasticity is dependent on exercise [235]. Furthermore, BDNF mediates microglial proliferation, promotes microglia and astrocyte activation and has potential to increase neuroinflammation [233]. It can be speculated that changes in thermoregulatory processes that were crucial for increasing the PALs of *Homo erectus* onwards may have come at an evolutionary cost, altering the expression of BDNF dependent microglia and consequentially that of proinflammatory CNS markers implicated in neuropsychiatric disorders [232]. Thus, there is evidence for a role of microglial cells in the evolution of neuropsychiatric disorders. Therefore, this view suggests that evolutionary psychiatry may become a novel and distinct area of research and doctrine in the field of biological psychiatry [236]. Due to the mismatch between prehistoric and modern environments, humans are becoming susceptible to inflammatory markers due to chronic stress. Evolutionary psychiatry offers an explanatory model for understanding modern psychiatric disorders and their evolutionary antecedents. Psychiatry has reached a critical point where it needs to develop therapeutic methods that integrate evolutionary thinking. This will be especially important in the advent of novel biotechnologies that may alter psychoneuroendocrinological mechanisms in ways that are beyond our current understanding.

## Abbreviations

AD: Affective disorders
ALS: Amyotrophic lateral sclerosis
APSM: Abnormal spindle-like microcephaly-associated protein
AMPA: (S)-2-amino-3-(3-hydroxy-5-methyl-4-isoxazole)-propionic acid
APOE: Apolipoprotein type E
ASDs: Autism spectrum disorders
ATP: Adenosine triphosphate
BDNF: Brain-derived neurotrophic factor
CXCL1: Fractalkine
CD: Cluster of differentiation
CNS: Central nervous system
CSF: Cerebrospinal fluid
DAT: Dementia of Alzheimer’s type
DRN: Dorsal raphe nucleus
DRD: Dopamine receptors
ECT: Electroconvulsive therapy
ER: Endoplasmic reticulum
HLA-DR: Human leukocyte antigen
IBA1: Ionized calcium-binding adaptor molecule 1
IFN-α: Interferon-alpha
IL: Interleukin
KYN: Kynurenine
LPS: Lipopolysaccharide
MHC: Major histocompatibility complex
MDD: Major depressive disorder
NMDAR: N-methyl-D-aspartate receptors
NO: Nitric oxide
NOX2: NADPH oxidase 2
NRCAM: Neuronal cell adhesion molecule
PAL: Physical activity level
PATHOS-D: Pathogen host defence
PFC: Prefrontal cortex
PD: Parkinson’s disease
PSA: Polysialic acid
PET: Positron emission tomography
PND: Postnatal day
PGE2: Prostaglandin E2
SN: Substantia nigra
SIGLEC11: Sialic acid-binding Ig like lectin 11
SIRT 2: Silent information regulator 2
SSRI: Selective serotonin reuptake inhibitors
SZ: Schizophrenia
TEE: Total energy expenditure
TGF-α: Transforming growth factor alpha
TH-1: T-helper cells 1
TH-2: T-helper cells 2
TNF-α: Tumor necrosis factor alpha
TSPO PET: Translocator protein positron emission tomography

## References

[1] Mosser CA, Baptista S, Arnoux I, Audinat E (2017). Microglia in CNS development: Shaping the brain for the future. Prog Neurobiol.

[2] Ben Achour SB, Pascual O (2010). Glia: the many ways to modulate synaptic plasticity. Neurochem Int.

[3] Walter L, Neumann H (2009). Role of microglia in neuronal degeneration and regeneration. Semin Immunpathol.

[4] Napoli I, Neumann H (2009). Microglial clearance function in health and disease. Neuroscience.

[5] Nakagawa Y, Chiba K (2014). Role of microglial M1/M2 polarisation in relapse and remission of psychiatric disorders and diseases. Pharmaceuticals.

[6] Na KS, Jung HY, Kim YK (2014). The role of pro-inflammatory cytokines in the neuro-transmission and neurogenesis in schizophrenia. Prog Neuro-Psychopharmacol Biol Psychiatry.

[7] Brites D, Fernandes A (2015). Neuroinflammation and depression: microglia activation, extravesicular microvesicles and microRNA dysregulation. Front Cell Neurosci.

[8] Mayhew J, Beart PM, Walker FR (2015). Astrocyte and microglial control of glutamatergic signaling: A primer on understanding the disruptive role of chronic stress. J Neuroendocrin.

[9] Howes OD, McCutcheon R (2017). Inflammation and the neural diathesis-stress hypothesis of schizophrenia: a reconceptualization. Transl Psychiatry.

[10] Lenz KM, McCarthy MM (2015). A starring role for microglia in brain sex differences. Neuroscientist.

[11] Orihuela R, McPherson CA, Harry GJ (2016). Microglial M1/M2 polarization and metabolic states. Br J Pharmacol.

[12] Karve IP, Taylor JM, Crack PJ (2016). The contribution of astrocytes and microglia to traumatic brain injury. Br J Pharmacol.

[13] Thibaut F (2017). Neuroinflammation: new vistas for neuropsychiatric research. Dialogues Clin Neurosci.

[14] De Picker LJ, Morrens M, Chance SA, Boche D (2017). Microglia and brain plasticity in acute psychosis and schizophrenia illness course: a meta-review. Front Psychiatry.

[15] Meyer U, Feldon J, Dammann O (2013). Schizophrenia and autism: Both shared and disorder-specific pathogenesis via perinatal inflammation. Prog Neuro-Psychopharmacol Biol Psychiatry.

[16] Lurie DI (2018). An integrative approach to neuroinflammation in psychiatric disorders and neuropathic pain. J Exper Neurosci.

[17] Najjar S, Pearlman DM (2015). Neuroinflammation and white matter pathology in schizophrenia: systematic review. Schizophr Res.

[18] Chew LJ, Fusar-Poli P, Schmitz T (2013). Oligodendrocyte alterations and the role of microglia in white matter injury: relevance to schizophrenia. Dev Neurosci.

[19] Doorduin J, de Vries EFJ, Willemsen ATM, de Groot JC, Dierck RA, Klein HC (2009). Neuroinflammation in schizophrenia-related psychosis: A PET study. J Nucl Med.

[20] Skaper SD, Facci L, Giusti P (2014). Neuroinflammation, microglia and mast cells in the pathophysiology of neurocognitive disorders: a review. CNS Neurol Disord Drug Targets.

[21] Filiou MD, Arefin AS, Moscato P, Graeber MB (2014). Neuroinflammation differs categorically from inflammation: transcriptomes of Alzheimer´s disease, Parkinson´s disease, schizophrenia and inflammatory diseases compared. Neurogenetics.

[22] Kim YK, Na KS, Myint AM, Leonard BE (2016). The role of pro-inflammatory cytokines in neuroinflammation and the neuroendocrine system in major depression. Prog Neuro-Psychopharmacol Biol Psychiatry.

[23] Mallya AP, Deutsch AY (2018). (Micro)Glia as effectors of cortical volume loss in schizophrenia. Schizophr Bull.

[24] Szepesi Z, Manouchehrian O, Bachiller S, Deierborg T (2018). Bidirectional microglia-neuron communication in health and disease. Front Cell Neurosci.

[25] Volk DW (2017). Role of microglia disturbances and immune-related marker abnormalities in cortical circuitry dysfunction in schizophrenia. Neurobiol Dis.

[26] Blank T, Prinz M (2013). Microglia as modulators of cognition and neuropsychiatric disorders. Glia.

[27] Bollinger JL, Wohleb ES (2019). The formative role of microglia in stress-induced synaptic deficits and associated behavioral consequences. Neurosci Lett..

[28] Zhao H, Alm A, Chen Q, Eusman MA, Pal A, Eguchi S (2017). The role of microglia in the pathobiology of neuropathic pain development: what do we know?. Br J Anasth.

[29] Uranova NA, Vikhreva OV, Rakhmanova VI, Orlovskaya DD (2020). Dystrophy of oligodendrocytes and adjacent microglia in prefrontal gray matter in schizophrenia. Front Psychiatry.

[30] Müller N, Myint AM, Schwarz MJ (2009). The impact of neuron in the dysregulation on neuroprotection and neurotoxicity in psychiatric disorders-relation to drug treatment. Dialog Clin Neurosci.

[31] Réus GZ, Fries GR, Stertz L, Badaway M, Passos IC, Barichello T, Kupczinski F, Quevedo J (2015). The role of inflammation and microglial activation in the pathophysiology of psychiatric disorders. Neuroscience.

[32] Bernstein HG, Steiner J, Guest PC, Dobrowolny H, Bogerts B (2015). Glial cells as key players in schizophrenia pathology. Schizophr Res.

[33] Leza JC, Garcia-Bueno B, Bioque M, Arango C, Parellada M, Do K, O’Donnell P, Bernardo M (2015). Inflammation in schizophrenia: A question of balance. Neuroscience Biobehav. Rev..

[34] Hong H, Kim BS, Im H-I (2016). Pathophysiological role of neuroinflammation in neurodegnerative diseases and psychiatric disorders. Int Neurolurol J.

[35] Tay TL, Bléchade C, D’Andrea I, St-Pierre MK, Henry MS, Roumier A, Tremblay ME (2018). Microglia gone rogue: impacts on psychiatric disorders across the lifespan. Front Mol Neurosci.

[36] Morgan JT, Chana G, Pardo CA, Achim C, Semendeferi K, Buckwalter J, Courchsese E, Everall IP (2010). Microglial activation and increased microglial density observed in the dorsolateral prefrontal cortex in autism. Biol Psychiatry.

[37] Tetreault NA, Hakeem AY, Jiang S, Williams BA, Allman E, Wold BJ, Allman JM (2012). Microglia in the cerebral cortex in autism. J Autism Dev Disord.

[38] Bilbo S, Stevens B (2017). Microglia: the brain’s first responders. Cerebrum.

[39] Racki V, Petric D, Kucic N, Grezeta N, Jurdana K, Roncevic-Grzeta I (2016). Cortical gray matter loss in schizophrenia: Could microglia be the culprit? Med. Hypotheses.

[40] Trépanier MO, Hopperton KE, Mizrahi R, Mechawar N, Bazinet RP (2016). Postmortem evidence of cerebral inflammation in schizophrenia: a systematic review. Mol Psychiatry.

[41] van Kesteren CFMG, Gremmels H, de Witte LD, Hol EM, Van Gool AR, Falkai PG, Kahn RS, Sommer IEC (2017). Immune involvement in the pathogenesis of schizophrenia: a meta-analysis on postmortem brain studies. Transl Psychiatry.

[42] Foster R, Kandanearatchi A, Beasley C, Williams B, Khan N, Fagerhol MF, Everall IP (2006). Calprotectin in microglia from frontal cortex is up-regulated in schizophrenia. Evidence for an inflammatory process*?*. Eur J Neurosci.

[43] Imai Y, Ibata I, Ito D, Ohsawa K, Kohsaka S (1996). A novel gene 1 in the major histocompatibility complex class III region encoding an EF hand protein expressed in a monocytic lineage. Biochem Biophys Res Commun.

[44] Petrasch-Parwez E, Schöbel A, Benali A, Moinfar Z, Förster E, Brüne M, Juckel G (2020). Lateralization of increased density of Iba 1-immunopositive microglial cells in the anterior midcingulate cortex of schizophrenia and bipolar density. Eur Arch Psychiatry Clin Neurosci.

[45] Bloomfield PS, Selvaraj S, Veronese M, Rizzo G, Bertoldo A, Owen DR, Bloomfield MAP, Bonoldi I, Kalk N, Turkheimer F, McGuire P, de Paola V, Howes OD (2016). Microglial activity in people at ultra risk of psychosis and in schizophrenia: An [^11^C] PBR28 PET brain imaging study. Am J Psychiatry.

[46] Hafizi S, Tseng HH, Rao N, Selvanathan T, Kenk M, Bazinet RP, Suridjan I, Wilson AA, Meyer JH, Remington G, Houle S, Rusjan PM, Mizrahi R (2017). Imaging microglial activation in untreated first-episode psychosis: a PET study with [^18^] FEPPA. Am J Psychiatry.

[47] Di Biase MA, Zalesky A, O’Keefe G, Laskaris L, Baune BT, Weickert CS, Olver J, McGorry PD (2017). PET imaging of putative microglial activation in individuals at ultra-high risk for psychosis, recently diagnosed and chronically ill with schizophrenia. Transl. Psychiatry.

[48] Marques TR, Ashok AH, Pillinger T, Veronese M, Turkheimer FE, Dazzan P, Sommer IEC, Howes OD (2019). Neuroinflammation in schizophrenia: meta-analysis of in vivo microglial imaging studies. Psychol Medicine.

[49] Conen S, Gregory CJ, Hinz R, Smallman R, Corsi-Zuelli F, Deakin B, Talbot PS (2020). Neuroinflammation as measured positron emission tomography in patients with recent onset and established schizophrenia: implications for immune pathogenesis. Mol Psychiatry.

[50] Banati R, Hickie IB (2009). Therapeutic signposts: using biomarkers to guide better treatment of schizophrenia. MJA.

[51] Burmester GR, Pezutto A (2003) Color atlas of immunology. Thieme Publishers Stuttgart New York. pp. 1–322

[52] Steiner J, Bielau H, Brisch R, Danos P, Ullrich O, Mawrin C, Bernstein HG, Bogerts B (2008). Immunological aspects in the neurobiology of suicide: Elevated microglial density in schizophrenia and depression is associated with suicide. J Psychiatry Res.

[53] Bayer TA, Buslei R, Havas L, Falkai P (1999). Evidence for activation of microglia in patients with psychiatric illnesses. Neurosci Lett.

[54] Radewicz K, Garey LJ, Gentleman S, Reynolds R (2000). Increase in HLA-DR immunoreactive microglia in frontal and temporal cortex of chronic schizophrenics. J Neuropathol Exp Neurol.

[55] Wierzba-Bobrowicz T, Lewandowska E, Lechowicz W, Stepien T, Pasenik E (2005). Qualitative analysis of activated microglia, ramified and damage of processes in the frontal and temporal lobes of chronic schizophrenics. Folia Neuropathol.

[56] Krause D, Wagner J, Matz J, Weidinger E, Obermeier M, Riedel M, Gruber R, Schwarz M, Mueller N (2012). Monocytic HLA DR antigens in schizophrenic patients. Neurosci Res.

[57] Steiner J, Mawrin C, Ziegeler A, Bielau H, Ullrich O, Bernstein HG, Bogerts B (2006). Distribution of HLA-DR-positive microglia in schizophrenia reflects impaired cerebral lateralization. Acta Neuropathol.

[58] Fillman SG, Cloonan N, Catts VS, Miller LC, Wong J, McCrossin T, Cairns M, Weickert CS (2013). Increased inflammatory markers identified in the dorsolateral prefrontal cortex of individuals with schizophrenia. Mol Psychiatry.

[59] Busse S, Busse M, Schiltz K, Bielau H, Gos T, Brisch R, Mawrin C, Schmitt A, Jordan W, Müller UJ, Bernstein HG, Bogerts B (2012). Different distribution patterns of lymphocytes and microglia in the hippocampus of patients with residual schizophrenia versus paranoid schizophrenia: Further evidence for disease course-related immune alterations. Brain Behav Imm.

[60] Sneeboer MAM, Snijders GJLJ, Berdowski WM, Fernandez-Andreu A, Psychiatric Donor Program of the Netherlands Brain Bank (NBB-Psych), van Mierlo HC, van Berlekom AB, Litjens M, Kahn RS, Hol EM, de Witte LD (2019) Microglia in post-mortem brain tissue of patients with bipolar disorder are not immune activated. Transl Psychiatry 9:153 10.1038/s41398-019-0490-x PMC6534632 31127084

[61] Shigeta Y, Ishii H, Takagi S, Yoshitake Y, Hirano T, Takata H (1980). HLA antigens as immunogenetic markers of alcoholism and alcoholic liver diseases. Pharmacol Biochem Beh.

[62] Cook RT, Gurvey MJ, Booth BM, Goeken JA, Stewart B, Noel M (1991). Activated CD-8 cells and HLA-DR expression in alcoholics without overt liver disease. J Clin Immunol.

[63] Laso FJ, Madruga JF, San Miguel JF, López M, Alvarez M, Orfao A (1996). Long lasting immunological effects of ethanol after withdrawal. Cytometry.

[64] Pan Y, Wang KS, Wang L, Wu LY (2013). Common variants in HLA-DRA gene are associated with alcohol dependence in two Caucasian samples. J Mol Neurosci.

[65] Laskaris LE, Biase MA, Everall I, Chana G, Christopoulos A, Skafidas E, Cropley VI, Pantelis C (2016). Microglial activation and progressive brain changes in schizophrenia. Br J Pharmacol.

[66] Al-Haddad BJS, Oler E, Armistead B, Elsayed NA, Weinberger DR, Bernier R, Burd I, Kapur R, Jacobsson B, Wang C, Mysorekar I, Rajagopal L, Waldorf KMA (2019). The fetal origins of mental illness. Am J Obstet Gynecol.

[67] Goudriaan A, de Leeuw C, Ripke S, Hultman CM, Sklar P, Sullivan PF, Smit AB, Posthuma D, Verheijen MHG (2014). Specific glial functions contribute to schizophrenia susceptibility. Schizophr Bull.

[68] Arinami T, Otsuka Y, Hamaguchi H, Itokawa M, Aoki J, Shibuya H, Okubo Y, Jwawaki Y, Ota K, Enguchi H, Tagaya H, Yano S, Shimuzu H, Torio M (1998). Evidence supporting an association between the DRB1 gene and schizophrenia in Japanese. Schizophr Res.

[69] Sasaki T, Matsushita M, Nanko S, Fukuda R, Kennedy JL, Tokunga K (1999). Schizophrenia and HLA-DRB1 gene in the Japanese population. Am J Psychiatry.

[70] Wright P, Nimgaonkar VL, Donaldson PT, Murray RM (2001). Schizophrenia and HLA: a review. Schizophr Res.

[71] Müller N, Ackenheil M (1998). Psychoneuroimmunology and the cytokine action in the CNS: implications for psychiatric disorders. Prog Neuropsychopharmacol Biol Psychiatry.

[72] Shi L, Fatemi SH, Sidwell RW, Patterson PH (2003). Maternal influenza infection causes marked behavioral and pharmacological changes in the offspring. J Neurosci.

[73] Cowan M, Petri WA (2018). Microglia: Immune regulators of neurodevelopment. Front Immunol.

[74] Jiang NM, Cowan M, Moonah SN, Petri WA (2018). The impact of systematic inflammation on neurodevelopment. Trends Mol Med.

[75] Nelson LH, Saulsbery AI, Lenz KM (2019). Small cells with big implications: microglia and sex differences in brain development, plasticity and behavioral health. Prog Neurobiol.

[76] Osborne BF, Turano A, Caulfield JI, Schwarz JM (2019). Sex- and region-specific differences in microglia phenotype and characterization of the peripheral immune response following early-life infection in male and female rats. Neurosci Lett.

[77] Smolders S, Smolders SMT, Swinnen N, Gärtner A, Rigo J-M, Legendre P, Brone B (2015). Maternal immune activation evoked by polyinosinic:polycytidylic acid does not evoke microglial cell activation in the embryo. Front Cell Neurosci.

[78] Juckel G, Manitz MP, Brüne M, Friebe A, Heneka MT, Wolf RJ (2011). Microglial activation in a neuroinflammational animal model of schizophrenia - a pilot study. Schizophr Res.

[79] Shapiro LA, Perez ZD, Foresti ML, Arisi GM, Ribak CE (2009). Morphological andultrastructural features of Iba1-immunolabeled microglial cells in the hippocampal dentate gyrus. Brain Res.

[80] Stence N, Waite M, Dailey ME (2001). Dynamics of microglial activation: a confocal time-lapse analysis in hippocampal slices. Glia.

[81] Manitz MP, Esslinger M, Wachholz S, Plümper J, Friebe A, Juckel G, Wolf RJ (2013). The role of microglia during life span in neuropsychiatric disease — an animal study. Schizophr Res.

[82] Buschert J, Sakalem ME, Saffari R, Hohoff C, Rothermundt M, Arolt V, Zhang W, Ambrée O (2016). Prenatal immune activation in mice blocks the effects of environmental enrichment on explatory behavior and microglia density. Prog Neuro-Psychopharmacol Biol Psych.

[83] Vidal PM, Pacheco R (2020). The cross-talk between the dopaminergic and the immune system involved in schizophrenia. Front Pharmacol.

[84] World Health Organization (2014) Preventing suicide: a global imperative. pp. 1–92

[85] Ernst C, Mechawar N, Turecki G (2009). Suicide neurobiology. Prog Neurobiol.

[86] American Psychiatric Association (2013). Diagnostic and statistical manual of mental disorders.

[87] Baker K, Halliday GM, Törk I (1990). Cytoarchitecture of the human dorsal raphe nucleus. J Comp Neurol.

[88] Stockmeier CA, Shapiro LA, Haycock JW, Thompson PA, Lowy MT (1996). Quantitative subregional distribution of serotonin_1A_ receptors and serotonin transporters in the human dorsale raphe. Brain Res.

[89] Valentino RJ, Commons KG (2005). Peptides that fine-tune the serotonin system. Neuropeptides.

[90] Lowry CA, Hale MW, Evans AK, Heerkens J, Staub DR, Gasser PJ, Shekhaw A (2008). Serotonergic systems, anxiety, and affective disorder: Focus on the dorsal raphe nucleus. Ann N Y Acad Sci.

[91] Ranft K, Dobrowolny H, Krell D, Bielau H, Bogerts B, Bernstein HG (2010). Evidence for structural abnormalities of the human habenular complex in affective disorders but not in schizophrenia. Psychol Med.

[92] Zhao H, Zhang BL, Yang SJ, Rusak B (2015). The role of lateral habenula-dorsal raphe nucleus circuits in higher brain functions and psychiatric illness. Behav Brain Res.

[93] Kasper JM, McCue DL, Milton AJ, Szwed A, Sampson CM, Huang M, Carlton S, Meltzer HY, Cunningham KA, Hornell JD (2016). Gamma-aminobutyric acidergic projections from the dorsal raphe to the nucleus accumbens are regulated by neuromedin U. Biol Psychiatry.

[94] Bolderini M, Underwood MD, Mann JJ, Arango V (2005). More tryptophan hydroxylase in the brainstem dorsal raphe nucleus in depressed suicides. Brain Res.

[95] Bonkale WL, Murdock S, Janosky JE, Austin MC (2004). Normal levels of tryptophan hydroxylase immunoreactivity in the dorsal raphe of depressed suicide victims. J Neurochem.

[96] Bielau H, Mawrin C, Krell D, Agelink MW, Trübner K, Davis R, Gos T, Bogerts B, Bernstein HG, Baumann B (2005). Differences in activation of the dorsal raphe nucleus depending on performance of suicide. Brain Res.

[97] Craven RM, Priddle TH, Coopert SJ, Crow TJ, Esiri MM (2005). The dorsal raphe nucleus in schizophrenia: a post-mortem study of 5-hydroxytryptamine neurons. Neuropathol Appl Neurobiol.

[98] Bonkale WL, Turecki G, Austin MC (2006). Increased tryptophan hydroxylase immunoreactivity in the dorsal raphe nucleus of alcohol-dependent, depressed suicide subjects is restricted to the dorsal subnucleus. Synapse.

[99] Bach-Mizrachi H, Underwood MD, Kassir SA, Bakalian MJ, Sibille E, Tamir H, Arango V, Mann J (2006). Neuronal tryptohan hydroxylase mRNA expression in the human dorsal and median raphe nuclei. Neuropsychopharmacol.

[100] Bach-Mizrachi H, Underwood MD, Tin A, Ellis SP, Mann JJ, Arango V (2008). Elevated expression of tryptophan hydroxylase-2 mRNA at the neuronal level. Mol Psychiatry.

[101] Gos T, Krell D, Brisch R, Bielau H, Trübner K, Steiner J, Bernstein HG, Baumann B (2008). Demonstration of decreased activity of dorsal raphe nucleus neurons in depressed suicidal patients by the AgNOR staining method. J Affect Disord.

[102] Krzyzanowska M, Steiner J, Karnecki K, Kaliszan M, Brisch R, Wiekowski M, Braun K, Jankowski Z, Gos T (2016). Decreased ribosomal DNA transcription in dorsal raphe differentiates between suicidal and non-suicidal death. Eur Arch Psychiatry Clin Neurosci.

[103] Krzyzanowska M, Steiner J, Brisch R, Mawrin C, Busse S, Karnecki K, Jankowski Z, Gos T (2016). Decreased ribosomal DNA transcription in dorsal raphe nucleus is specific for suicide regardless of psychiatric diagnosis. Psychiatry Res.

[104] Brisch R, Steiner J, Mawrin C, Krzyzanowska M, Jankowski Z, Gos T (2017). Microglia in the dorsal raphe nucleus plays a potential role in both suicide facilitation and prevention in affective disorders. Eur Arch Psychiatry Clin Neurosci.

[105] Bach H, Arango V, Kassir SA, Tsaava TT, Dwork AJ, Mann J, Underwood MD (1997). Alcoholics have more tryptophan hydroxylase 2 mRNA and protein in the dorsal and median raphe nuclei. Alcohol Clin Exp Res.

[106] Stockmeier CA (1997). Neurobiology of serotonin in depression and suicide. Ann N Y Acad Sci.

[107] Jasinska AJ, Lowry CA, Burmester M (2012). Serotonin transporter gene, stress and raphe-raphe interactions: a molecular mechanism of depression. Trends Neurosci.

[108] Albert PR, Benkelfast C (2013) The neurobiology of depression-revisiting the serotonin hypothesis. II Genetic, epigenetic, and clinical studies. Phil Trans R Soc B 368:20120535 10.1098/rstb.2012.0535 PMC3638388 23440469

[109] Mann JJ (2013). The serotonergic system in mood disorders and suicidal behavior. Phil Trans R Soc B.

[110] Hahn A, Haeusler D, Kraus C, Höflich AS, Kranz GS, Baldinger P (2014). Attenuated serotonin transporter association between dorsal raphe and ventral striatum in major depression. Hum Brain Mapping.

[111] Bobillier P, Seguin S, Petitjean F, Salvert D, Touret M, Jouvet M (1976). The raphe nuclei of the rat brain stem: a topographical atlas of their efferent projections as revealed by autoradiography. Brain Res.

[112] Soares JC, Mann J (1997). The functional neuroanatomy of mood disorders. J Psychiatr Res.

[113] Underwood MD, Khaibulina AA, Ellis SP, Moran A, Rice PM, Mann J, Arango V (1999). Morphometry of the dorsal raphe nucleus serotonergic neurons in suicide victims. Biol Psychiatry.

[114] Arango V, Underwood MD, Boldrini M, Tamir H, Kassir SA, Hsiung SC, Chen JJ, Mann JJ (2001). Seotonin 1A receptors, serotonin transporter binding and serotonin transporter mRNA expression in the brainstem of depressed suicide victims. Neuropsychopharmacol.

[115] Nestler EJ, Barrot M, DiLeone RJ, Eisch AJ, Gold SJ (2002). Neurobiology of depression. Neuron.

[116] Hornung JP (2003). The human raphe nuclei and the serotonergic system. J Chem Neuroanatomy.

[117] Mann JJ (2003). Neurobiology of suicidal behavior. Nature Rev Neurosci.

[118] Nemeroff CB, Vale WW (2005). The neurobiology of depression: in roads to treatment and new drug discovery. J Clin Psychiatry.

[119] Bolderini M, Underwood MD, Mann JJ, Arango V (2008). Serotonin-1A autoreceptor binding in the dorsal raphe nucleus of depressed suicides. J Psychiatry Res.

[120] Matthews PR, Harrison PJ (2012). A morphometric, immunohistochemical, and in situ hybridization study of the dorsal raphe nucleus in major depression, bipolar disorder, schizophrenia, and suicide. J Affect Disord.

[121] Quesseveur G, Reperant C, David DJ, Gardier AM, Sanchez C, Guiard BP (2013). 5-HT_2A_ receptor inactivation potentiates the acute antidepressant-like activity of escitalopram: involvement of the noradrenergic system. Exp Brain Res.

[122] Challis C, Berton O (2015). Top-down control of serotonin systems by the prefrontal cortex: a path toward restored socioemotional in depression. ALS Chem Neurosci.

[123] Rahn KA, Cao YJ, Hendrix CW, Kaplin AI (2015). The role of 5-HT1A receptors in mediating acute negative effects of antidepressants: implications in pediatric depression. Transl Psychiatry.

[124] Sullivan GM, Oquendo MA, Milak JM, Miller JM, Burke A, Ogden RT, Parsey RV, Mann JJ (2015). Positron emission tomography quantification of serotonin_1A_ receptor binding in suicide attempters with major depressive disorder. JAMA Psychiat.

[125] Dankoski EC, Carroll S, Wightman RM (2016). Acute selective serotonin reuptake inhibitors regulate the dorsal raphe nucleus causing amplification of terminal serotonin release. J Neurochem.

[126] Wang L, Zhou C, Zhu D, Wang X, Fang L, Zhong J, Mao Q, Sun L, Gong X, Xia J, Lian B, Xie P (2016). Serotonin-1A receptor alterations in depression: a meta-analysis of molecular imaging studies. BMC Psychiatry.

[127] Malone KM, Waternaux C, Haas GL, Cooper TB, Li S, Mann J (2003). Cigarette smoking, suicidal behavior, and serotonin function in major psychiatric disorders. Am J Psychiatry.

[128] Linker KE, Elabd MG, Tawadrous P, Cano M, Green KN, Wood MA, Leslie FM (2020). Microglial activation increases cocaine self-administration following adolescent nicotine exposure. Nat Commun.

[129] Wachholz S, Eßlinger M, Plümper J, Manitz MP, Juckel G, Friebe A (2016). Microglia activation is associated with IFN-α induced depressive-like behavior. Brain Behav Imm.

[130] Schnieder TP, Trencevska I, Rosoklija G, Stankov A, Mann J, Smiley J, Dwork AJ (2014). Microglia of prefrontal white matter in suicide. J Neuropathol Exp Neurol.

[131] Suzuki H, Ohgidani M, Kuwano N, Chrétien F, Lorin de la Grandmaison G, Onaya M, Tominaga I, Setoyama D, Kang D, Mimura M, Kanba S, Kato TH (2019). Suicide and microglia: recent findings and future perspectives based on human studies. Front Cell Neurosci.

[132] Mallya AP, Wang HD, Lee HNR, Deutsch AY (2019). Microglial pruning of synapses in the prefrontal cortex during adolescence. Cereb Cortex.

[133] Jawaid A, Krajewska J, Pawliczak F, Kandra V, Schulz PE (2016). A macro role for microglia in poststroke depression. JAGS.

[134] Singhal G, Baune T (2017). Microglia: an interface between the loss of neuroplasticity and depression. Front Cell Neurosci.

[135] Serafini G, Pompili M, Seretti ME, Stefani H, Palermo M, Coryell W, Girardi P (2013). The role of inflammatory cytokines in suicidal behavior: A systematic review. Eur Neuropsychopharmacol.

[136] Mina VAL, Lacerda-Pinheiro SF, Maia LC, Pinheiro RFF, Meireles CB, de Souza SIR, Reis AOA, Bianco B, Rolim MLN (2015). The influence of inflammatory cytokines in physiopathology of suicidal behavior. J Affect Disord.

[137] Gananca L, Oquendo MA, Tyrka AR, Cisneros-Trujilo S, Mann JJ, Sublette ME (2016). The role of cytokines in the pathophysiology of suicidal behavior. Psychoneuroendocrinology.

[138] Torres-Platas SG, Cruceanu C, Chen GG, Turecki G (2014). Evidence for increased microglial priming and macrophage recruitment in the dorsal anterior cingulate white matter of depressed suicides. Brain Behav Immun.

[139] Slusarczyk J, Trojan E, Glombik K, Budziszewska B, Kubera M, Lason W, Papiolek-Barczyl K, Mika J, Wedzong K, Basta-Kaim A (2015). Prenatal stress is a vulnerability factor for altered morphology and biological activity of microglial cells. Front Cell Neurosci.

[140] Stein DJ, Vasconcelos MF, Albrecht-Souza L, Ceresér KMM, de Almeida RMM (2017). Microglial over-activation contributes to anxiety- and depressive-like behaviors. Front Behav Neurosci.

[141] Turano A, Lawrence JH, Schwarz JM (2017). Activation of neonatal microglia can be influenced by other neural cells. Neurosci Lett.

[142] Matcovitch-Natan O, Winter DR, Giladi A, Aguilar SV, Spinrad A, Sarrazin S, Ben-Yehuda H, David E, González FZ, Perrin P, Keren-Shaul H, Gury M, Lara-Astaiso D, Thaiss CA, Cohen M, Halpern KB, Baruch K, Deczkowska A, Lorenzo-Vivas E, Itzkovitz S, Elinav E, Sieweke MH, Schwartz M, Amit I (2016). Microglia development follows a stepwise program to regulate brain homeostasis. Science.

[143] O’Loughin E, Pakan JM, Yilmazer-Hanke D, McDermott KW (2017). Acute in utero exposure to lipopolysaccharide induces inflammation in the pre- and postnatal brain and alters the glial cytoarchitecture in the developing amygdala. J. Neuroinflamm..

[144] Polazzi E, Monti B (2010). Microglia and neuroprotection: From in vitro studies to therapeutic applications. Prog Neurobiol.

[145] Hetman M, Pietrzak M (2012). Emerging roles of the neuronal nucleolus. Trends Neurosci.

[146] Parlatto R, Kreiner G (2013). Nucleolar activity in neurodegenerative diseases: a missing piece of the puzzle?. J Mol Med.

[147] Yirmiya R, Rimmerman N, Reshef R (2015). Depression as a microglial disease. Trends Neurosci.

[148] Bray JC, Reyes KC, Roberts AJ, Ransohoff RM, Gruol DL (2013). Synaptic plasticity in the hippocampus shows resistance to acute ethanol exposure in transgenic mice with astrocyte-targeted enhanced CCL2 expression. Neuropharmacol.

[149] Tian L, Hui CW, Bisht K, Tan Y, Sharma K, Chen S, Zhang X, Tremblay M-E (2017). Microglia under psychosocial stressors along the aging trajectory: consequences on neuronal circuicts, behavior, and brain diseases. Prog Neuro-Psychopharmacol Biol Psychiatry.

[150] Prata J, Santos SG, Almeida MI, Coelho R, Barbosa MA (2017). Bridging autism spectrum disorders and schizophrenia through inflammation and biomarkers – pre-clinical and clinical investigations. J Neuroinflammation.

[151] Neumann J, Gunzer M, Gutzeit HO, Ullrich O, Reymann KG, Dinkel K (2006). Microglia provide neuroprotection after ischemia. FASEB.

[152] Neumann H, Kotter MR, Franklin RJM (2009). Debris clearance by microglia: an essential link between degeneration and regeneration. Brain.

[153] Biber K, Neumann H, Inoue K, Boddecke HW (2007). Neuronal on and off signals control microglia. Trends Neurosci.

[154] Kettenmann H, Hanisch UK, Noda M, Verkhratsky A (2011). Physiology of microglia. Physiol Rev.

[155] DiSabato DJ, Quan N, Godbout JP (2016). Neuroinflammation: the devil is in the detail. J Neurochem.

[156] Sellgren CM, Sheridan SD, Gracias J, Xuan D, Fu T, Perlis RH (2017). Patient-specific models of microglia-mediated engulfment of synapses and neural progenitors. Mol Psychiatry.

[157] Takahashi Y, Yu Z, Sakai M, Tomita H (2016). Linking activation of microglia and peripheral monocytic cells to the pathophysiology of psychiatric disorders. Front Cell Neurosci.

[158] Hui CW, St.-Pierre A, El Hajj H, Remy Y, Hebert S, (2018). Prenatal immune challenge in mice leads to partly sex-dependent behavioral, microglial, and molecular abnormalities associated with schizophrenia. Front Mol Neurosci.

[159] Hinwood M, Morandini J, Day TA, Walker FR (2012). Evidence that microglia mediate the neurobiological effects of chronic psychological stress on the medial prefrontal cortex. Cereb Cortex.

[160] Hinwood M, Tynan RJ, Charnley JL, Beynon SB, Day TA, Walker FR (2013). Chronic stress induced remodeling of the prefrontal cortex: structural re-organization of microglia and the inhibitory effect of minocycline. Cereb Cortex.

[161] Wohleb ES, Delpech JC (2016). Dynamic cross-talk between microglia and peripheral monocytes underlies stress-induced neuroinflammation and behavioral consequences. Prog-Neuropsychopharmacol Biol Psychiatry.

[162] Müller N, Myint A-M, Schwarz MJ (2009). The impact of neuroimmunedysregulation on neuroprotection and neurotoxicity in psychiatric disorders-reaction to drug treatment. Dial Clin Neurosci.

[163] Müller N, Myint A-M, Schwarz MJ (2012). Inflammation in schizophrenia. Adv Protein Chem Struct Biol.

[164] Jo WK, Zhang Y, Emrich HM, Dietrich DE (2015). Glia in the cytokine-mediated onset of depression: fine tuning the immune response. Front Cell Neurosci.

[165] Bagasrawala I, Zecevic N, Radonjic NV (2016). N-methyl D-aspartate receptor antagonist kynurenic acid affects human cortical development. Front Neurosci.

[166] Kegel ME, Bhat M, Skogh E, Samuelsson M, Lundberg K, Dahl ML, Sellgren C, Schwieler L, Engberg G, Schuppe-Kiostinen I, Erhardt S (2014). Imbalanced kynurenine pathway in schizophrenia. Int J Tryptophan Res.

[167] Larsson MK, Schwieler L, Goiny M, Erhardt S, Engberg G (2015). Chronic antipsychotic treatment in the rat-effects on brain interleukin-8 and kynurenic acid. Int J Tryptophan Res.

[168] Erhardt S, Schwieler L, Imbeault S, Engberg G (2017). The kynurenine pathway in schizophrenia and bipolar disorder. Neuropharmacology.

[169] Notarangelo FM, Pocivavsek A (2017). Elevated kynurenine pathway metabolism during neurodevelopment: implications for brain and behavior. Neuropsychopharmacology.

[170] Pitman E, Iwata Y, Caravaggio F, Nakajima S, Chung JK, Gerretsen P, Kim J, Takeuchi H, Chakravarty MM, Remington G, Graff-Guerro A (2017). Kynurenic acid in schizophrenia: a systematic review and meta-analysis. Schizophr Bull.

[171] Wurfel BE, Drevets BE, Bliss SA, McMillin JR, Suzuki H, Ford BN, Morris HM, Teague TK, Dantzer R, Savitz JB (2017). Serum kynurenic acid is reduced in affective psychosis. Transl. Psychiatry.

[172] Brundin L, Erhardt S, Bryleva EY, Achtyes ED, Postolache TT (2011). The role of inflammation in suicidal behavior. Acta Psychiatr Scand.

[173] Müller N, Myint AM, Krause D, Weidinger E, Schwarz MJ (2013). Anti-inflammatory treatment in schizophrenia. Prog Neuro-Psychopharmacol Biol Psychiatry.

[174] Steiner J, Walter M, Gos T, Guillemin GJ, Bernstein HG, Sarnyai Z, Mawrin C, Brisch R, Bielau H, Meyer zu Schwabendissen L, Bogerts B, Myint AM (2011). Severe depression is associated with increased quinolinic acid in subregions of the anterior cingulate gyrus. Evidence for an immune-modulated glutamatergic neurotransmission?. J Neuroinflamm..

[175] Busse M, Busse S, Myint AM, Gos T, Dobrowolny H, Müller UJ, Bogerts B, Bernstein HG, Steiner J (2015). Decreased quinolinic acid in the hippocampus of depressive patients: evidence for local anti-inflammatory and neuroprotective responses?. Eur Arch Psychiatry Clin Neurosci.

[176] Steiner J, Bogerts B, Sarnyai Z, Walter M, Gos T, Bernstein HG, Myint AM (2012). Bridging the gap between the immune and glutamate hypotheses of schizophrenia and major depression: Potential role of glial NMDA receptor modulators and impaired blood-brain barrier integrity. World J Biol Psychiatry.

[177] Kubesova A, Tejkalova H, Syslova K, Kacer P, Vondrousova J, Tyls F, Fujakova M, Palenicek T, Horacek J (2015). Biochemical, histopathological and morphological profiling of a rat model of early immune stimulation: relation to psychopathology. PLoS ONE.

[178] Kato TA, Monji A, Mizoguchi Y, Hashioka S, Horikawa H, Seki Y (2011). Anti-inflammatory properties of antipsychotics via microglia modulations. Are antipsychotics a fire extinguisher in the brain of schizophrenia?. Mini Rev Med Chem.

[179] Miller BJ, Buckley P, Seabolt W, Mellor A, Kirkpatrick B (2011). Meta-analysis of cytokine alterations in schizophrenia: Clinical status and antipsychotic effects. Biol Psychiatry.

[180] Mizoguchi Y, Kato T, Horikawa H, Monji A (2014). Microglial intracellular Ca^2+^ signaling as a target of antipsychotic actions for the treatment of schizophrenia. Front Cell Neurosci.

[181] Hashimoto K (2008). Microglial activation in schizophrenia and minocycline treatment. Prog Neuro-Psychopharmacol Biol Psychiatry.

[182] Hashioka S, McGeer PL, Miyaoka T, Wake R, Horiguchi J (2015). Can inhibition of microglial activation cure schizophrenia. Schizophr Res.

[183] Giovanoli S, Engler H, Engler A, Richetto J, Feldon J, Riva MA, Schedlowski M, Meyer U (2016). Preventive effects of minocycline in a neurodevelopmental two-hit model with relevance to schizophrenia. Transl. Psychiatry.

[184] Riazi K, Galic MA, Kentner AC, Reid AY, Sharkey KA, Pittman QJ (2015). Microglia-dependent alteration of glutamatergic synaptic transmission and plasticity in the hippocampus during peripheral inflammation. J Neurosci.

[185] Kobayashi K, Imagama S, Ohgomori T, Hirano K, Uchimuru K, Sakamoto K, Herakawa A, Takeuchi H, Suzumura A, Ishiguro N, Kordomatsu K (2013). Minocycline selectively inhibits M1 polarization of microglia. Cell Dise.

[186] Cotel MC, Lenartowicsz EM, Nateasan S, Modo MM, Cooper JD, Williams SCR, Kapur S, Vernon AC (2015). Microglial activation in the rat brain following chronic antipschotic treatment at clinically relevant doses. Eur Neuropsychopharmacol.

[187] Ribeiro BMM, Santos do Carno MR, Souze Freire R, Flavio N, Rocha, M, Borella VCM, (2013). Evidences for a progressive microglial activation and increase in iNOS expression in rats submitted to a neurodevelopmental model of schizophrenia: Reversal by clozapine. Schizophr Res.

[188] Kenk M, Selvanathan T, Rao N, Suridan I, Rusjan P, Remington G, Meyer JH, Wilson AA, Houle S, Mizrahi R (2015). Imaging neuroinflammation in gray and white matter in schizophrenia: An in-vivo PET study with [^18^F]-FEPPA. Schizophr Bull.

[189] Monji A, Kato T, Kanba S (2009). Cytokines and schizophrenia: microglia hypothesis of schizophrenia. Psychiatry Clin Neurosci.

[190] Frick LR, Williams K, Pittenger C (2013). Microglial dysregulation in psychiatric disease. Clin Dev Immunol.

[191] Inta D, Lang UE, Borgwardt S, Meyer-Lindenberg A, Gass P (2017). Microglia activation and schizophrenia: Lessons from the effects of minocycline on postnatal neurogenesis, neuronal survival and synaptic pruning. Schizophr Bull.

[192] Monji A, Kato TA, Mizoguchi Y, Horikawa H, Seki Y, Kasai M, Yamauchi Y, Yamada S, Kanba S (2013). Neuroinflammation in schizophrenia especially focused on the role of microglia. Prog Neuro-Psychopharmacol Biol Psychiatry.

[193] van Rees GF, Lago SG, Cox DA, Tomasi KJ, Rustogi N, Weigelt K, Ozcan S, Cooper J, Drexhage H, Leweke FM, Bahn S (2018). Evidence of microglial activation following exposure to serum from first-onset drug-naïve schizophrenic patients. Brain Behav Immun.

[194] Bhattacharya A (2018). Recent advances in CNS P2X7 physiology and pharmacology: focus on neuropsychiatric disorders. Front Phamacol.

[195] Dong XH, Zhen XC (2015). Glial Pathology in bipolar disorder. CNS Neurosci Ther.

[196] Stertz L, Magalhaes PVS, Kapczinski F (2013). Is bipolar disorder an inflammatory condition: the relevance of microglial activation. Curr Opn Psychiatry.

[197] Zhang F, Zhou H, Wilson BC, Shi JS, Hong JS, Gao HM (2012) Fluoxetine protects against microglial activation-mediated neurotoxicity. Parkinsonism Relat Disord 18S1:S213-S217 10.1016/S1353-8020(11)70066-9 PMC3608679 22166439

[198] Tynan RJ, Weidenhofer J, Hinwood M, Cairns MJ, Day TA, Walker FR (2012). A comparative examination of the anti-inflammatory effects of SSRI and SNRI antidepressants on LPS stimulated microglia. Brain Beh Imm.

[199] Horikawa H, Kato TA, Mizoguchi Y, Monji A, Seki Y, Ohkuri T, Gotoh L, Yonaha M, Ueda T, Hashioka S, Kanba S (2010). Inhibitory effects of SSRI on IFN-y induced microglial activation through the regulation of intracellular calcium. Prog Neuro-Psychopharmacol Biol Psychiatry.

[200] Dhami KS, Churchward MA, Baker GB, Todd KG (2019). Fluoxetine and its metabolite norfluoxetine induce microglial apoptosis. J Neurochem.

[201] Leonard BE (2014). Impact of inflammation on neurotransmitter changes in major depression. An insight into the action of antidepressants. Prog Neuro-Psychopharmacol Biol Psychiatry.

[202] Dubovicky M, Csazar E, Melichercikova K, Kuniakova M, Rackova L (2014). Modulation of microglial function by the antidepressant drug venlafaxine. Interdiscip Toxicol.

[203] Takeuchi H (2010). Neurotoxicity by microglia: Mechanisms and potential therapeutic strategy. Clin Exper Neuroimmunol.

[204] Palmieri EM, Menga A, Lebrun A, Hooper DC, Butterfield DA, Mazzone M, Castegna A (2017). Blockade of glutamine synthetase enhances inflammatory response in microglial cells. Antioxid Redox Signal.

[205] Parellada E, Gasso P (2021) Glutamate and microglia activation as a driver of dendritic apoptosis: a core pathophysiological mechanism to understand schizophrenia. Trans Psychiatry 10.1038/s41398-021-01385-9 10.1038/s41398-021-01385-9 PMC8102516 33958577

[206] Ohgidani M, Kato TA, Kanba S (2015). Introducing directly induced microglia-like (iMG) cells from fresh human monocytes: a novel translational research tool for psychiatric disorders. Front Cell Neurosci.

[207] Prytkova I, Brennand KJ (2017). Prospects for modeling abnormal neuronal function in schizophrenia using human induced pluripotent stem cells. Front Cell Neurosci.

[208] Gomes FV, Llorente R, Delbel EA, Viveros MP, López-Gallardo M, Guimarães FS (2015). Decreased glial reactivity could be involved in the antipsychotic-like effect of cannabiol. Schizophr Res.

[209] Lisboa SF, Gomes FV, Guimaraes FS, Campos AC (2016). Microglial cells as a link between cannabinoids and the immune hypothesis of psychiatric disorders. Front Neurol.

[210] de Almeida V, Martins-de-Souza D (2018). Cannabinoids and glial cells: possible mechanism to understand schizophrenia. Eur Arch Psychiatry Clin Neurosci.

[211] Cortez IL, Rodrigues da Silva NR, Guimaraes FS, Gomes FV (2020) Are CB2 receptors a new target for schizophrenia treatment. Front. Psychiatry 11:587154 10.3389/fpsyt.2020.587154 PMC7673393 33329132

[212] Jinno S, Kosaka T (2008). Reduction of Iba1-expressing microglial process density in the hippocampus following electroconvulsive shock. Expr Neurol.

[213] Jansson L, Wennström M, Johanson A, Tingström A (2009). Glial cell activation in response to electroconvulsive seizures. Prog Neuropsychopharmacol Biol Psychiatry.

[214] Limoa E, Hashioka S, Miyaoka T, Tsuchie K, Arauchi R, Azis IA, Wake R, Hayashida M, Araki T, Furuya M, Liaury K, Tanra AJ (2016). Electroconvulsive shock attenuated microgliosis and astrogliosis in the hippocampus and ameliorated schizophrenia-like behavior of Gunn rat. J Neuroinflamm.

[215] Hartenstein V, Giangrande A (2018). Connecting the nervous and the immune systems in evolution. Comm Biol.

[216] Schlegelmilch T, Henke K, Peri F (2011). Microglia in the developing brain: From immunity to behaviour. Curr Opin Neurobiol.

[217] Swinnen N, Smolders S, Avila A, Notelaers K, Paesen R, Ameloot M (2013). Complex invasion pattern of the cerebral cortex by microglial cells during development of the mouse embryo. Glia.

[218] Verkhratsky A, Ho MS, Parpura V, Verkhratsky A, Ho MS, Zorec R, Parpura V (2019). Evolution of neuroglia. Neuroglia in Neurodegenerative Diseases.

[219] Haug H (1987). Brain sizes, surfaces and neuronal sizes of the cortex cerebri: A stereological investigation of man and his variability and a comparison with some mammals (primates, whales, marsupials, insectivores and one elephant). Am J Anat.

[220] Evans PD, Anderson JR, Vallender EJ, Gilbert SL, Malcom CM, Dorus S (2004). Adaptive evolution of ASPM, a major determinant of cerebral cortical size in humans. Hum Mol Genet.

[221] Martín-Loeches M (2010). Uses and abuses of the enhanced-working-memory hypothesis in explaining modern thinking. Curr Anthropol.

[222] Hayakawa T, Angata T, Lewis AL, Mikkelsen TS, Varki NM, Varki A (2005). A human-specific gene in microglia. Science.

[223] Wang Y, Neumann H (2010). Alleviation of neurotoxicity by microglial Human Siglec-11. J Neurosci.

[224] Raison CL, Miller AH (2013). The evolutionary significance of depression in Pathogen Host Defence (PATHOS-D). Mol Psychiatry.

[225] Panter-Brick C (2002). Sexual division of labor: energetic and evolutionary scenarios. Am J Hum Biol.

[226] Brisch R, Saniotis A, Wolf R, Bielau H, Bernstein HG, Steiner J, Bogerts B, Braun K, Jankowski Z, Kumaratilake J, Henneberg M, Gos T (2014). The role of dopamine in schizophrenia from a neurobiological and evolutionary perspective: old fashioned, but still in vogue. Front Psychiatry.

[227] Tyler H, Polk JD (2019). BDNF, endurance activity, and mechanisms underlying the evolution of hominin brains. Am J Phys Anthropol.

[228] Ruxton GD, Wilkinson DM (2011). Avoidance of overheating and selection for both hair loss and bipedality in hominins. Proc Nat Acad Sci.

[229] Mattson MP, Wan R (2008). Neurotrophic factors in autonomic nervous system plasticity and dysfunction. NeuroMol Med.

[230] Noakes T, Spedding M (2012). Run for your life. Nature.

[231] Raichlen DA, Polk JD (2013). Linking brains and brawn: Exercise and the evolution of human neurobiology. Proc R Soc B Biol Sci.

[232] Saniotis A, Henneberg M (2013). Evolutionary medicine and future humanity: will evolution have the final word?. Humanities.

[233] Ding H, Chen J, Su M (2020). BDNF promotes activation of astrocytes and microglia contributing to neuroinflammation and mechanical allodynia in cyclophosphamide-induced cystitis. J Neuroinflamm.

[234] Lipsky RH, Marini AM (2007). Brain-derived neurotrophic factor in neuronal survival and behavior-related plasticity. Ann N Y Acad Sci.

[235] Rasmussen P, Brassard P, Adser H, Pedersen MV, Leick L, Hart E, Secher NH, Pedersen BK, Pilegaard H (2009). Evidence for a release of brain-derived neurotrophic factor from the brain during exercise. Exper Physiol.

[236] Saniotis A (2019). Evolutionary psychiatry enhancing our current knowledge of psychopathologies. Arch Psychiatry Psychotherapy.

